# Genome Sequence of Novel Clostridium perfringens Bacteriophage vB_CpeS-17DYC, Isolated from Wastewater in China

**DOI:** 10.1128/mra.00300-23

**Published:** 2023-06-20

**Authors:** Amina Nazir, Qing Zhang, Yuqing Liu, Yibao Chen

**Affiliations:** a Shandong Key Laboratory of Animal Disease Control and Breeding, Institute of Animal Science and Veterinary Medicine, Shandong Academy of Agricultural Sciences, Jinan, China; b Key Laboratory of Livestock and Poultry Multi-omics of MARA, Shandong Academy of Agricultural Sciences, Jinan, China; Loyola University Chicago

## Abstract

Phage vB_CpeS-17DYC was isolated from wastewater from a poultry market using Clostridium perfringens strain DYC. The vB_CpeS-17DYC genome is 39,184 bp long, with 65 open reading frames and a GC content of 30.6%. It shared 93.95% nucleotide identity, with 70% query coverage, with *Clostridium* phage phiCP13O (GenBank accession number NC_019506.1). Virulence factor genes were not found in the vB_CpeS-17DYC genome.

## ANNOUNCEMENT

A variety of systemic and enteric diseases have been attributed to Clostridium perfringens, including health care-associated infections. Humans and animals are thus exposed to serious health risks from these bacteria (1). Infections caused by these antibiotic-resistant bacteria are becoming an increasing concern that could be treated with phage therapy (2, 3).

Phage vB_CpeS-17DYC was isolated from wastewater in Jinan, China (36°40′13″N, 117°01′15″E), on 31 March 2023. C. perfringens strain DYC was used as the bacterial host for the isolation of phage. In brief, a water sample was homogenized with modified Gifu anaerobic medium (GAM) broth for 60 s, inoculated with 100 μL of bacterial culture, centrifuged at 12,000 × *g* at 4°C for 20 min, and filtered through a sterile membrane filter with a pore size of 0.45 μm. Phage dilutions were mixed with host cultures, poured into molten top agar at 55°C, spilled onto plates of tryptic soy agar, and incubated anaerobically at 37°C for 24 h. A visible plaque was picked up and further purified with the help of serial dilution using saline magnesium buffer (1).

Phage DNA was extracted using the phenol-chloroform method and sequenced. Following the use of 100 ng of DNA with the NEBNext Ultra II FS DNA library preparation kit, DNA was quantified using a Qubit fluorometer (New England Biolabs). DNA fragmentation was performed along with amplification and purification via PCR and AMPure XP beads, respectively. The Agilent 2100 Bioanalyzer system was utilized to detect scattering of library fragments with KAPA library quantification kits. Complete genome sequencing of phage vB_CpeS-17DYC was performed using the NovaSeq 6000 system (Illumina, San Diego, CA, USA) with the 2 × 150-bp paired-ended format. Adapter sections and low-quality reads were removed by means of Trimmomatic v0.36 (4). SOAPdenovo v0.36 was used to assemble the sequences (5). The phage vB_CpeS-17DYC genome was annotated using RAST (6) and BLASTp with the National Center for Biotechnology Information (NCBI) nonredundant database (7). tRNAscan-SE v2.0 was used to look for potential tRNA genes (8). VirulenceFinder (9) and ResFinder (10) were used to identify putative virulence factor genes and antibiotic resistance genes, respectively. Default parameters were used for all software.

Phage vB_CpeS-17DYC possesses a circular double-stranded genome of 39,184 bp (1,539,339 total reads), with a GC content of 30% and an average read coverage of 947.6×. A total of 65 potential genes were predicted for the genome of vB_CpeS-17DYC, but no tRNA gene was detected. The phage vB_CpeS-17DYC genome did not contain any predicted virulence factor or drug resistance genes. According to the NCBI search with BLASTn v2.13.0 (last accessed 17 April 2023), the vB_CpeS-17DYC phage shared 93.95% nucleotide identity, with 70% query coverage, with *Clostridium* phage phiCP13O (GenBank accession number NC_019506.1). After the BLASTp search, 39 genes in the phage genome were assigned putative functions, while the products of 26 genes remained hypothetical proteins. The following gene modules were identified: (i) structural and packaging proteins (tail proteins, major capsid protein, and terminase large subunit), (ii) metabolism and replication (exonuclease and DNA polymerase), (iii) lysis (holin and *N*-acetylmuramoyl-l-alanine amidase), and (iv) hypothetical proteins. The open reading frame 50 (ORF50) protein functions as an antirepressor protein; the antirepressor protein promotes the viral life cycle by neutralizing the inhibitory effect of the repressor protein, thus enabling the phage to take over the host bacterial cell and replicate (11).

This article does not contain any studies with human participants or animals performed by any of the authors.

### Data availability.

The genome sequence of phage vB_CpeS-17DYC is available in GenBank under accession number OQ717002, with NCBI SRA accession number SRR24076211 and BioSample accession number SAMN33960761.
